# Supplementary material for: Combining motor imagery with action observation training does not lead to a greater autonomic nervous system response than motor imagery alone during simple and functional movements: a randomized controlled trial
Source: PeerJ. 2018 Jul 6;6:e5142. doi: 10.7717/peerj.5142 (PMC6037142; doi:10.7717/peerj.5142)
Supplement: Supplemental Information 3 [file peerj-06-5142-s003.docx]

| 1. Título de la investigación: Evaluación de la respuesta del Sistema Nervioso Simpático con el entrenamiento de Observación de Acciones e Imaginería Motora.   PROTOCOLO PARA LA VALORACIÓN DE PROYECTOS DEL COMITÉ ÉTICO DE INVESTIGACIÓN.  Línea de investigación a la que se adscribe:  Responsable de la misma: Roy La Touche Arbizu |  |
| --- | --- |
| 1. Nombre y Apellidos del Investigador principal: (adjuntar CV)   Nombre y apellidos: Roy La Touche Arbizu  Teléfono: 654949149  Email: roylatouche@lasallecampus.es  DNI: | |
| 1. Facultad/departamento del que depende el investigador principal.   Facultad de Ciencias de la Salud del Centro Superior de Estudios Universitarios La Salle, Departamento de Fisioterapia. | |
| 1. Nombre y Apellidos del resto de investigadores (adjuntar CV)   Investigador 1: Ferran Cuenca Martínez  Teléfono: 628936505  Email: Fcuema@irflasalle.es  DNI: 20455212R | |
| 1. Facultad/departamento del que dependen el resto de investigadores.   Facultad de Ciencias de la Salud del Centro Superior de Estudios Universitarios La Salle, Departamento de Fisioterapia. | |
| 1. Institución o entidad donde se llevará a cabo la investigación   Laboratorio del Centro Superior de Estudios Universitarios La Salle. | |
| 1. Entidad que financia el proyecto   No procede | |
| 1. Fecha inicio: Enero 2017     Fecha finalización: Septiembre 2017 | **:** |

| 1. Antecedentes, estado del tema anterior a la investigación.(máximo 12 líneas)   La Imaginería Motora (IM) se define como un estado mental dinámico durante el cual, la representación de una acción se realiza de manera interna sin ninguna ejecución real motora(1,2). Por otro lado, la Observación de Acciones (OA) simula mentalmente los movimientos que el observador visualmente está percibiendo(3). Ambos procesos mentales provocan una activación de las áreas corticales relacionadas con la planificación y ejecución motora, de manera muy similar, que cuando la acción se realiza de manera real(4–7). Se ha demostrado que la IM y la OA provocan cambios en el Sistema Nervioso Autónomo (SNA) causando respuestas simpático excitatorias, aunque las bases neurofisiológicas siguen siendo inciertas y basadas en hipótesis(8–10).  Pero no solamente existen ensayos clínicos que demuestren la activación y participación del SNA durante la práctica mental sino también hay estudios como el realizado por Mulder y su grupo donde no obtuvieron datos satisfactorios con respecto a las respuestas del SNA y los procesos mentales(11,12). | | |
| --- | --- | --- |
| 1. Justificación: razones y motivación por las que se realiza el estudio. Exponer brevemente el estado actual del problema planteado.(máximo 15 líneas)   En base a la literatura actual, parece coexistir cierta disparidad en relación a los resultados que se han obtenido con respecto a la implicación de la actividad del SNA y los procesos de IM y OA. Además, ningún ensayo clínico ha cuantificado de manera tangible y objetiva dicha actividad durante los procesos de la práctica mental limitándose exclusivamente a hacer un análisis cualitativo basado en actividad o no actividad del SNA. La participación de variables autonómicas simpático excitatorias es de interés potencialmente relevante debido a que aporta una interfaz entre práctica mental y ejecución real de una acción debido a la posible correlación de una mayor actividad del mismo, mayor similitud de respuestas neurofisiológicas entre imaginación y observación con respecto a la realidad de la acción motora. | | |
| 1. Hipótesis y objetivos.   Hipótesis alternas:  Nuestra hipótesis reside en que el entrenamiento de OA y de IM provoca una activación del SNA simpático excitatorio mayor que la IM de manera aislada y donde a mayor grado de activación y participación del mismo, existe mayor similitud entre los procesos neurofisiológicos que acontecen tanto a la práctica mental como en la ejecución real de una ejecución motora.  Por tanto, existirán diferencias estadísticamente significativas con respecto a la activación del SNA simpático excitatorio en el grupo que combina la IM con el entrenamiento en OA frente al grupo entrenado solo con IM. Del mismo modo, se producirá una mayor activación en aquellos sujetos que presenten mayor capacidad para imaginar y mayor nivel de actividad física.  Hipótesis nula:  El entrenamiento en OA junto con la terapia de IM no presentará diferencias estadísticamente significativas con respecto a la activación del SNA simpático excitatorio frente al grupo entrenado solo con IM. Del mismo modo, no se producirá una mayor activación en aquellos sujetos que presenten mayor capacidad para imaginar y mayor nivel de actividad física.  Objetivos:  Principales:  • Evaluar y cuantificar los cambios simpáticos producidos por la técnica de IM y el entrenamiento de OA en el instrumento de medidas de variables autonómicas en sujetos sanos en comparación con los producidos solamente por la técnica de IM.  • Evaluar la efectividad de la IM y de la IM junto con el entrenamiento de OA con respecto a la capacidad de imaginar.  Secundarios:    • Evaluar la relación entre el grado de activación del SNA simpático excitatorio en relación al nivel de actividad física.  • Evaluar la capacidad de la IM y de la IM junto con el entrenamiento de OA empleadas sobre variables de carácter psicosocial (grado de Autoeficacia y Kinesiofobia). | | |
| 1. Diseño metodológico: carácter cuantitativo o cualitativo, diseño de la investigación, modalidad de estudio, método de aleatorización.   Se llevará a cabo un ensayo clínico aleatorio controlado. La aleatorización se realizará mediante el programa informático GraphPad Software.  Los participantes del estudio se distribuirán en dos grupos:   - Un grupo con sujetos sanos que cumplan los criterios de inclusión y exclusión establecidos para el estudio, al cual se le realizará la técnica de Observación de Acciones, y posteriormente, de Imaginería Motora al tiempo que se registrarán los cambios simpáticos excitatorios provocados por las mismas. - Un grupo con sujetos sanos que cumplan los criterios de inclusión y exclusión establecidos para el estudio, al cual se le realizará la técnica de Imaginería Motora de manera aislada, registrando al mismo tiempo los cambios simpáticos excitatorios provocados por la misma. | | |
| 1. Sistema de selección de la población y muestra.(Indicar reclutamiento de la muestra)   -Se mandarán correos a los alumnos del Centro Universitario de La Salle.  -Se colocarán carteles informativos en las instalaciones del Centro Universitario de La Salle destinados a los alumnos, personal docente y administrativo, así como en distintos barrios de la Comunidad de Madrid. | | |
| - Criterios para la inclusión/ exclusión de participantes en la investigación.   Criterios de inclusión:   - Sujetos sanos con edades comprendidas entre 18 y 65 años. - No estar tomando medicación durante el periodo del estudio. - No tener ninguna condición dolorosa. - Sujetos sin historia previa de patología cardio-respiratoria. - Hablar y comprender el idioma español, siendo capaces de responder a preguntas verbales, cuestionarios y entender las instrucciones durante los procedimientos del estudio.   Criterios de exclusión:   - Sujetos que presenten patología sistémica, cardio-respiratoria, del Sistema Nervioso Central o enfermedad reumática así como cualquier patología musculo-esquelética. - Sujetos menores de edad. - Sujetos que no se encuentren en pleno uso de sus facultades mentales para poder realizar la intervención del estudio.   Eliminación:   - Aparición de dolor en el pecho o en cualquier otra región del tórax o abdomen durante la realización del estudio. - Falta de asistencia del sujeto a la cita prevista para la realización del estudio. - Aparición de trastornos psicológicos graves durante la realización del estudio. | | |
| 1. Razones del uso o ausencia de grupos de control, incluyendo la justificación para placebo. “Intervención o procedimiento sobre el grupo control y/o experimental (máximo 15 líneas)”   El grupo control de sujetos asintomáticos incluido en el estudio, se realizará con el objetivo de poder comparar los resultados de dicho grupo y comprobar la eficacia de las técnicas utilizadas basándonos en la hipótesis de que la previa observación de una acción se facilita la posterior imaginación de la misma.  El grupo control por tanto, tiene el objetivo poder comparar los resultados obtenidos, y por consiguiente, comprobar la eficacia de la práctica mental en relación a la activación del sistema simpático excitatorio. En primer lugar se le entregará a los sujetos el consentimiento informado. Posteriormente deberán rellenar una serie de cuestionarios para evaluar las variables incluidas en el estudio. Una vez completada esta primera fase se realizará las técnicas de OA + IM o IM dependiendo del grupo de estudio y posteriormente se procederá a la recogida de datos mediante un instrumento de medición de cambios simpáticos excitatorios. | | |
| 1. Tratamiento del grupo control   El grupo control realizará una intervención mediante IM exclusivamente. | |  |
| 1. Instrumentos utilizados para la obtención y recolección de información relacionada con el objeto de estudio   Para la cuantificación y evaluación de las variables autonómicas simpático excitatorias, siendo estas, la conductancia de la piel, la frecuencia cardíaca, la frecuencia respiratoria, presión arterial y la temperatura de la piel, se utilizará el instrumento de medición de cambios autonómicos MP 150 *System* de la empresa BIOPAC *Systems*, INC.  Para la evaluación de las variables secundarias se utilizarán:   - Escala de Autoeficacia   La autoeficacia mediante la versión corta de la Escala General de Autoeficacia, la cual evalúa la iniciativa, la persistencia y el esfuerzo. Se trata de una escala validada al Español y ha demostrado validez, fiabilidad y una buena consistencia interna(13).   - Kinesiofobia o Miedo al movimiento   El miedo al movimiento o Kinesiofobia se cuantificará con la versión española de la Escala Tampa de Kinesiophobia (TSK-11). Este instrumento demuestra fiabilidad y validez en pacientes con dolor crónico, siendo más breve que la escala original. La puntuación total oscila entre 11 y 44 puntos, siendo las puntuaciones más altas las que indican mayor temor a una nueva lesión debido al movimiento(14).   - Cuestionario Internacional de Actividad Física (IPAQ)   Se utilizará el cuestionario IPAQ validado y traducido al Español. El objetivo del cuestionario IPAQ es obtener datos relacionados con actividad física relacionada con salud. Se utilizará la versión larga formada por 5 objetivos de actividad evaluados independientemente que incluyen preguntas acerca de la actividad física relacionada con el trabajo, con el transporte, con el trabajo en el hogar, con el tiempo libre y finalmente con el tiempo dedicado a estar sentado. Los resultados finales sugieren que estas medidas tienen aceptables propiedades de medición y son apropiadas para estudios nacionales poblacionales de prevalencia de participación en actividad física(15).   - Cuestionario de Imagen del Movimiento (MIQ-R)   El MIQ-R es un cuestionario con el objetivo de evaluar la capacidad de imaginación de los sujetos, el cual, está formado por ocho ítems, cuatro para una escala visual, y los otros cuatro para una escala cinestésica.  La puntuación de cada ítem del MIQ-R se efectúa en una escala de 7 puntos, donde el 1 indica “muy difícil de ver/sentir”, y el 7 indica “muy fácil de ver/sentir”.  La versión española del cuestionario MIQ-R tiene una consistencia interna alta y se correlaciona significativamente con otras medidas de imaginación de un movimiento u acción, así como con otras medidas que evalúan la claridad y agudeza de la capacidad de imaginar(16). | | |
| 1. Descripción de la naturaleza y grado de los riesgos que pudieran, previsiblemente, derivarse de la participación en la investigación.   El entrenamiento en OA y la terapia con IM no producirá riesgo asociados en los participantes del estudio. | | |
| 1. Previsión para evaluar y controlar eventualidades que pudieran suponer consecuencias para la salud presente y futura de los participantes en la investigación y/o de otras personas que pudieran verse afectadas por la investigación o sus resultados.   En la investigación no se realizará ninguna intervención que pueda suponer riesgos en la salud de los participantes. No obstante, los procedimientos que se llevarán a cabo se aplicarán en un entorno controlado para que el sujeto esté relajado y pueda desarrollar las actividades pautadas. | | |
| 1. Consentimiento informado para los participantes en la investigación (adjuntar modelo a la petición).   El consentimiento informado para los participantes incluidos en la investigación está adjuntado a la petición en el anexo I. | | |
| 1. Material visual o de otro tipo que vaya a ser utilizado en la investigación, solicitud de consentimiento específico. Sólo aplicable en los casos en los que se va a grabar de alguna manera las intervenciones que se realizan. (adjuntar modelo a la solicitud)   No procede. | | |
| 1. En el caso de personas no capaces de consentir, autorización para la participación de la investigación (adjuntar modelo a la solicitud)   No procede, todos los participantes serán mayores de edad con lo que todos son capaces de consentir participar y autorizar su propia participación en el estudio. | | |
| 1. Método para asegurar el respeto a la vida privada de los participantes en la investigación y de la confidencialidad de los datos personales.   Se garantiza la confidencialidad, el tratamiento, la comunicación y la cesión de los datos de carácter personal de todos los sujetos participantes se ajustará a lo dispuesto según la legislación sobre protección de datos vigente en España (Ley de Protección de Datos de Carácter Personal, 15/1999, de 13 de diciembre). | | |
| 1. Método para el tratamiento de la información que pudiera generarse durante la investigación. Comunicación de los resultados a los participantes y sus familias en el caso de ser relevante para su salud, presente o futura.   Los datos recogidos para el estudio estarán identificados mediante un código (número de sujeto) y solo los investigadores del estudio podrán relacionar dichos datos a los sujetos del estudio y relacionarlos con su historia clínica. Por lo tanto, su identidad no será revelada a ninguna persona.  Sólo se transmitirán a terceros y a otros países los datos recogidos para el estudio que en ningún caso contendrán información que le pueda identificar directamente, como nombre y apellidos, iniciales, dirección, número de la seguridad social, etc. En el caso de que se produzca esta cesión será para los mismos fines del estudio descrito y garantizando la confidencialidad como mínimo con el nivel de protección de la legislación vigente en nuestro país.  El acceso a la información personal quedará restringido a los investigadores, al Comité Ético de Investigación Clínica y personal autorizado por el investigador, cuando lo precisen para comprobar los datos y procedimientos del estudio, pero siempre manteniendo la confidencialidad de los mismos de acuerdo a la legislación vigente.    Los datos necesarios estarán guardados en nuestra base de datos que solo se podrán disponer de ellos en la Facultad de Ciencias de la Salud del Centro Universitario La Salle.  Se mandara una hoja informativa por correo a los participantes comunicando los resultados de la investigación, su resultado final y la fecha en la que serán expuestos dichos resultados. | | |
| 1. Plan de atención especializada derivada de la investigación tras la realización del proyecto, acceso al mismo de los participantes en la investigación. (Aplicable, por ejemplo, al grupo control si ha habido mejoras por la intervención en el grupo experimental)   Una vez finalizado el proyecto y teniendo en cuenta las características del tipo de estudio, no se realizará ningún seguimiento a los participantes del mismo. No obstante, se les proporcionará una dirección de correo electrónico a la cual podrán dirigirse en caso de que tengan alguna consecuencia derivada debido a la intervención. | | |
| 1. Detalle de los emolumentos y compensaciones propuestos por participar en la investigación.   La participación en este estudio es voluntaria y, por tanto, los participantes no recibirán ninguna compensación económica. | | |
| 1. Detalle de las circunstancias que podrían conducir a conflicto de interés y que podrían afectar al juicio independiente de los investigadores.   Debido a la ausencia de inversores y empresas que financien el presente estudio, no habrá problema de conflicto de interés. | | |
| 1. Detalle de cualquier uso futuro potencial, incluyendo el comercial, de los resultados de investigación, de otros datos recogidos durante el desarrollo del proyecto, o de muestras biológicas.   Se espera que los datos obtenidos con la realización de este proyecto puedan tener uso potencial en la práctica clínica en el momento de pautar tratamientos basados en la OA combinada con la IM, consiguiendo hacer progresiones más efectivas. Los investigadores del estudio creen que este estudio puede tener una repercusión en el mundo de la Fisioterapia, pero es posible que esto no ocurra. | | |
| 1. Detalle de la existencia de cualquier compensación, pago o bienes que sean proporcionados al investigador o su institución a cambio de la investigación.   El presente proyecto no tiene ningún tipo de interés económico, ni hay empresas o entidades que financien el estudio. Por lo tanto, al no haber ninguna fuente de recursos y ser un estudio voluntario para los sujetos que participen en él, no hay presente ningún emolumento para los investigadores responsables del mismo. | | |
| 1. Otras consideraciones éticas, que los autores de la investigación consideren oportuno indicar   Durante la realización del estudio, los investigadores se comprometen a trabajar con la máxima diligencia posible sin coaccionar a los sujetos participantes del mismo. Por otra parte, se dejará a los sujetos total libertad para exponer las posibles preguntas e inquietudes sobre las características teóricas y prácticas implicadas. Asimismo, los investigadores intentarán resolver las dudas que surjan con el máximo rigor científico, adaptando las respuestas al nivel de compresión de cada uno de los sujetos. | | |
| 1. Procedimientos para la compensación por daños que surjan en el contexto del proyecto de investigación   Durante la realización del estudio, se espera no causar ningún daño a los participantes que participen en el mismo. En caso de que se produzca algún daño derivado de este, se abordará de forma individualizada valorando la gravedad de los mismos e influencia que ha tenido el estudio para el agravamiento del estado del sujeto, y, en consecuencia se consensuará una forma de compensación que en ningún caso será de tipo económica. No obstante, el investigador principal está cubierto por un seguro de responsabilidad civil del Colegio de Fisioterapeutas. | | |
| 1. Bibliografía utilizada.   Referencias Bibliográficas:  1. Decety J. The neurophysiological basis of motor imagery. Behav Brain Res. 1996;77(1-2):45–52.  2. Frenkel M, Herzig D, Gebhard F, Mayer J, Becker C, Einsiedel T. Mental practice maintains range of motion despite forearm immobilization: A pilot study in healthy persons. J Rehabil Med. 2014 Mar;46(3):225–32.  3. Buccino G. Action observation treatment: a novel tool in neurorehabilitation. Philos Trans R Soc B Biol Sci. 2014 Apr 28;369(1644):20130185–20130185.  4. Lotze M, Montoya P, Erb M, Hülsmann E, Flor H, Klose U, et al. Activation of Cortical and Cerebellar Motor Areas during Executed and Imagined Hand Movements: An fMRI Study. J Cogn Neurosci. MIT Press 238 Main St., Suite 500, Cambridge, MA 02142-1046 USA journals-info@mit.edu ; 1999 Sep;11(5):491–501.  5. Stephan KM, Fink GR, Passingham RE, Silbersweig D, Ceballos-Baumann AO, Frith CD, et al. Functional anatomy of the mental representation of upper extremity movements in healthy subjects. J Neurophysiol. 1995 Jan;73(1):373–86.  6. Luft AR, Skalej M, Stefanou A, Klose U, Voigt K. Comparing motion- and imagery-related activation in the human cerebellum: a functional MRI study. Hum Brain Mapp. 1998;6(2):105–13.  7. Bunno Y, Suzuki T, Iwatsuki H. Motor imagery muscle contraction strength influences spinal motor neuron excitability and cardiac sympathetic nerve activity. J Phys Ther Sci. Society of Physical Therapy Science; 2015 Dec;27(12):3793–8.  8. Beyer L, Weiss T, Hansen E, Wolf A, Seidel A. Dynamics of central nervous activation during motor imagination. Int J Psychophysiol. 1990 Jul;9(1):75–80.  9. Lang PJ, Greenwald MK, Bradley MM, Hamm AO. Looking at pictures: affective, facial, visceral, and behavioral reactions. Psychophysiology. 1993 May;30(3):261–73.  10. Thill EE, Bryche D, Poumarat G, Rigoulet N. Task-involvement and ego-involvement goals during actual and imagined movements: their effects on cognitions and vegetative responses. Behav Brain Res. 1997 Jan;82(2):159–67.  11. Mulder T, de Vries S, Zijlstra S. Observation, imagination and execution of an effortful movement: more evidence for a central explanation of motor imagery. Exp Brain Res. Springer-Verlag; 2005 Jun 15;163(3):344–51.  12. Wuyam B, Moosavi SH, Decety J, Adams L, Lansing RW, Guz A. Imagination of dynamic exercise produced ventilatory responses which were more apparent in competitive sportsmen. J Physiol. Wiley-Blackwell; 1995 Feb 1;(Pt 3):713–24.  13. Herrero R, Espinoza M, Molinari G, Etchemendy E, Garcia-Palacios A, Botella C, et al. Psychometric properties of the General Self Efficacy-12 Scale in Spanish: general and clinical population samples. Compr Psychiatry. 2014 Oct;55(7):1738–43.  14. Gómez-Pérez L, López-Martínez AE, Ruiz-Párraga GT. Psychometric Properties of the Spanish Version of the Tampa Scale for Kinesiophobia (TSK). J Pain. 2011 Apr;12(4):425–35.  15. Roman-Viñas B, Serra-Majem L, Hagströmer M, Ribas-Barba L, Sjöström M, Segura-Cardona R. International Physical Activity Questionnaire: Reliability and validity in a Spanish population. Eur J Sport Sci. 2010;10(5):297–304.  16. Campos A, González MÁ. Spanish version of the revised movement image questionnaire (MIQ-R): Psychometric properties and validation. 2010;19(2):265–75. |  | |
| Anexos:  documentación aportada. | Consentimiento informado.  x  Consentimiento audio-visual.  Permiso no capaces consentir.  CV de los investigadores.  Permiso o aceptación de un centro colaborador para recoger en su sede las muestras necesarias o datos de la historia clínica de sus pacientes. 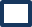  Otros: ………………………………………………….. | |
